# Feasibility of Guided Internet-Based Cognitive Behavioral Therapy for Panic Disorder and Social Anxiety Disorder in Japan: Pilot Single-Arm Trial

**DOI:** 10.2196/53659

**Published:** 2024-02-29

**Authors:** Seina Shinno, Kazuki Matsumoto, Sayo Hamatani, Yosuke Inaba, Yoshihito Ozawa, Yohei Kawasaki, Tomoki Ikai, Chihiro Sutoh, Hiroyuki Hayashi, Eiji Shimizu

**Affiliations:** 1 Department of Integrated Medical Sciences Graduate School of Medicine University of Fukui Fukui Japan; 2 Division of Clinical Psychology Kagoshima University Medical and Dental Hospital Kagoshima Japan; 3 Research Center for Child Mental Development Chiba University Chiba Japan; 4 Research Center for Child Mental Development University of Fukui Fukui Japan; 5 Biostatistics Section Clinical Research Center Chiba University Hospital Chiba Japan; 6 Faculty of Medical Sciences Division of Medicine Primary Health Care University of Fukui Fukui Japan; 7 Department of Emergency and General Medicine Fukui University Hospital Fukui Japan; 8 Department of Cognitive Behavioral Physiology Graduate School of Medicine Chiba University Chiba Japan

**Keywords:** cognitive behavioral therapy, internet intervention, panic disorder, social anxiety, feasibility trail, adult, adults, anxiety disorder, internet-based, e-learning, Japan, statistical analyses, therapist, therapists, intervention, severity, symptoms, therapeutic alliance, mobile phone

## Abstract

**Background:**

Cognitive behavioral therapy (CBT) is effective in treating anxiety disorders. Accessibility to CBT has been limited in Japan due to the shortage of therapists. While an open-source e-learning system can be used to create a simple internet-based cognitive behavioral therapy (ICBT) program, the safety and outpatient acceptance of this treatment approach have not been explored in Japan.

**Objective:**

The aim of this study was to investigate whether outpatients with anxiety disorders could accept and successfully complete the ICBT program with guidance by CBT therapists when implementing therapeutic modules and CBT tasks. Due to being in the initial phase of a novel treatment in Japan, this study was intended for verification with a small sample size.

**Methods:**

In total, 6 adults, including 4 male participants and 2 female participants, were enrolled in a single-arm trial. The intervention involved guided ICBT comprising 12 sessions, including CBT text, comprehension confirmation tests, and explanatory videos about cognitive behavioral models, accessible through a website. The therapist guided the participants in accessing the ICBT program and answering their questions using a chat tool. The primary outcome was anxiety severity assessed using the State-Trait Anxiety Inventory-Trait. Secondary outcomes included the Panic Disorder Severity Scale, Liebowitz Social Anxiety Scale (LSAS), Beck Anxiety Inventory (BAI), Patient Health Questionnaire–9, Generalized Anxiety Disorder–7, and Working Alliance Inventory–Short Form (WAI-SF). Statistical analyses were performed using paired 2-tailed *t* tests to assess the changes in clinical symptoms. The total WAI-SF score at the final session was used to evaluate the therapeutic alliance. For statistical analyses, mean changes for total State-Trait Anxiety Inventory-Trait, BAI, Panic Disorder Severity Scale, LSAS, Patient Health Questionnaire–9, and Generalized Anxiety Disorder–7 scores were analyzed using the paired 2-tailed *t* test. The 2-sided significance level for hypothesis testing was set at 5%, and 2-sided 95% CIs were calculated.

**Results:**

Most participants diligently engaged with the ICBT program. No adverse events were reported. The mean total scores for the primary outcome decreased by 11.0 (SD 9.6) points (95% CI –22.2 to 0.20; Hedges *g*=0.95), but it was not statistically significant. The mean total scores for the secondary outcomes that assess clinical symptoms decreased, with a significant reduction observed in the BAI of 15.7 (SD 12.1) points (95% CI –28.4 to –3.0; *P*=.03; Hedges *g*=1.24). The mean total scores for PDSS and LSAS decreased significantly, by 12.0 (SD 4.24) points (95% CI –50.1 to 26.1; *P*=.16; Hedges *g*=1.79) and 32.4 (SD 11.1) points (95% CI –59.7 to –4.3; *P*=.04; Hedges *g*=1.38), respectively. Of the participants, 67% (n=4) showed treatment response, and 50% (n=3) achieved remission after the intervention. The therapeutic alliance, measured using the WAI-SF, was moderate.

**Conclusions:**

Guided ICBT may be feasible for the treatment of outpatients with panic disorder and social anxiety disorder in Japan.

**Trial Registration:**

University Hospital Medical Information Network Clinical Trials Registry UMIN0000038118; https://center6.umin.ac.jp/cgi-open-bin/ctr/ctr_view.cgi?recptno=R000043439

## Introduction

Anxiety disorders such as social anxiety disorder (SAD) and panic disorder (PD) involve prolonged and significant anxiety, leading to substantial impairment in daily functioning. In Japan, the 12-month prevalence of anxiety disorders is 5.3%, making them the most commonly diagnosed mental disorders [1]. Cognitive behavioral therapy (CBT) has been proven to be effective in treating anxiety disorders, particularly SAD and PD [2,3]. As per the clinical guidelines [4,5], CBT is the primary treatment option for patients with SAD or PD. However, CBT has rarely been integrated into the Japanese psychiatric clinical practice. The limited availability of CBT therapists and high implementation costs have been major obstacles significantly impacting the accessibility of CBT [6,7]. According to a 2018 report, the implementation rate of CBT in Japanese psychiatric clinics was 6.2% [7]. When CBT is applied, it is rarely based on an evidence-based cognitive behavioral model. In most CBT practices, therapists independently select and combine CBT techniques [7].

Since the latter half of the 1990s, self-help CBT programs have been provided in rural areas via the internet. CBT delivered through the internet is known as internet-based cognitive behavioral therapy (ICBT) [8]. In the ICBT approach, therapists guide patients via phone or email to enhance their treatment experience. Previous studies of guided ICBT have demonstrated significant therapeutic effects on anxiety disorders. According to 2 systematic reviews with meta-analyses, guided ICBT is likely as effective as face-to-face CBT [9,10]. Most studies of guided ICBT in these reviews were conducted in Europe, particularly, Northern Europe [10-13]. Due to the varying internet infrastructure and ICT literacy across different countries, evidence from clinical trials in Eastern countries is crucial.

Recently, several studies on ICBT with treatment programs adapted to specific cultures have been conducted in China, Korea, and Pakistan, yielding results supporting the effectiveness of ICBT [14-16]. Although some culturally adapted ICBT programs for mental disorders have begun to emerge in Japan [17-20], there is a lack of evidence of clinical trials that specifically target anxiety disorders [21]. A previous single-arm study in Japan demonstrated the feasibility of CBT via videoconference as another remote format for 20 adults with SAD or PD [22]. In this study, remote therapists provided individual CBT in real time. The results indicated that Japanese individuals with anxiety disorders find remote interventions preferable and readily acceptable. However, in the guided ICBT format, the absence of face-to-face interaction with the therapist is fundamental, and it is anticipated that the treatment experience may qualitatively differ, potentially leading to less favorable patient acceptance and treatment responses.

To carefully assess the feasibility of guided ICBT, a novel treatment in Japan, we conducted a rigorously controlled clinical trial with a small sample size, monitoring for the risk of deterioration and unexpected adverse events. The participants’ guided ICBT experiences were also summarized in a brief case series included as Multimedia Appendix 1.

## Methods

### Study Design

This prospective single-arm open trial was conducted between September 2019 and March 2020 at 2 university hospitals and a psychiatric clinic in Japan. Patients with PD or SAD were recruited between September and November 2019 through posters and leaflets placed at medical institutions in the 2 prefectures and on the university’s home page. The participants continued to receive treatment from general medical practitioners during the study period, and their authorization was obtained before enrollment. Personal IDs and passwords to the treatment program and chat tools were provided to participants.

### Ethical Considerations

This study project and the present clinical trial protocol adhered to the Ethical Guidelines for Medical and Biological Research Involving Human Subjects in Japan [23]. This study was approved by the institutional review board of Chiba University Hospital (G2019004). Written informed consent was obtained from all participants after they were thoroughly informed of the study protocol. No compensation was provided for participating in the research, although the guided ICBT was provided for free. This pilot single-arm trial was registered at the University Hospital Medical Information Network Clinical Trials Registry (UMIN0000038118).

### Recruitment

**Figure 1:**
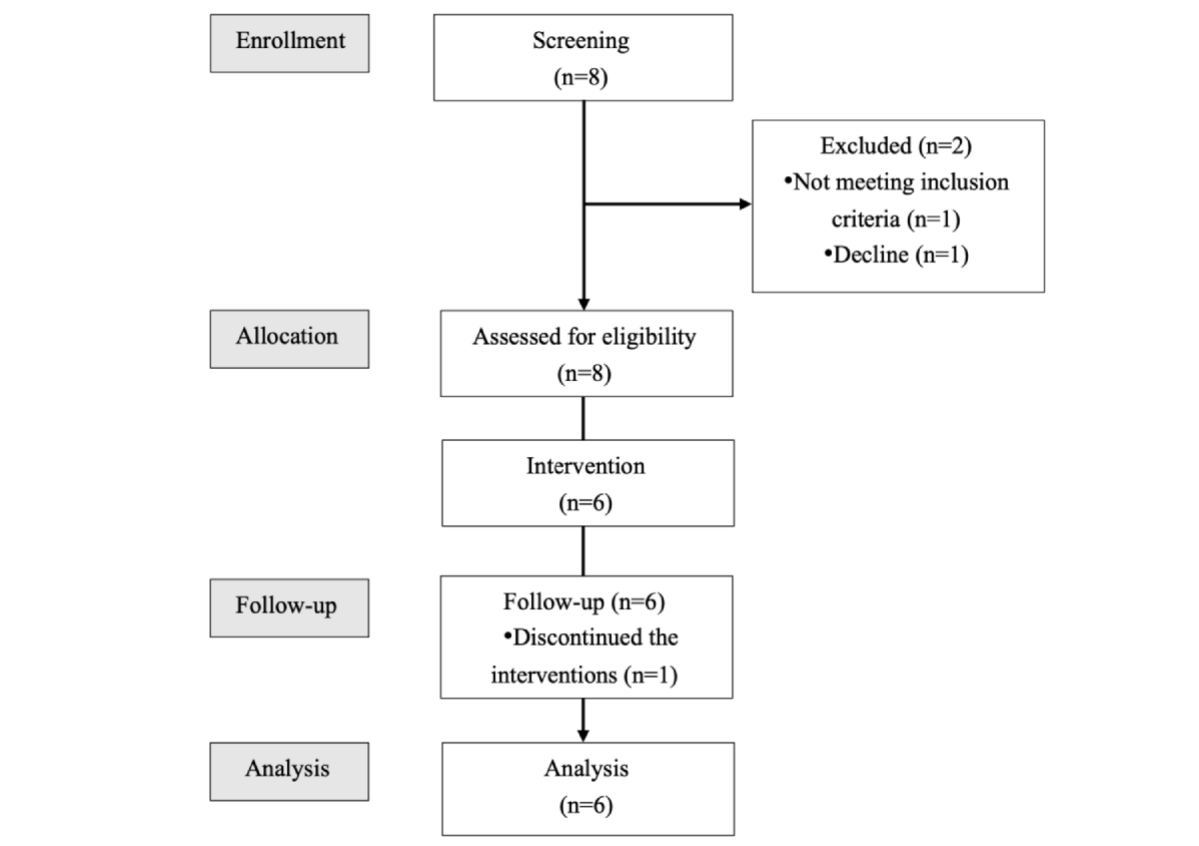
Participant flow.

Figure 1 illustrates the flow of participants throughout the study. Initially, 8 patients were recruited in the study. The screening through psychological assessments was conducted by 3 clinical psychologists (SS, KM, and SH), and it was ultimately confirmed by 2 physicians (HH and ES) whether the participants met the diagnostic criteria of the *Diagnostic and Statistical Manual of Mental Disorder, Fifth Edition* [24]. After the screening, 2 patients were excluded either because they did not meet the inclusion criteria or because of pending cancellations as part of the participant management process. In total, 6 patients underwent a baseline assessment and were subsequently enrolled in the study. Following enrollment, 1 patient disagreed with the exposure in session 6 during the intervention. Notably, there were no dropouts in this trial, and no adverse events were reported during treatment. Of the 6 patients, 5 (83%) successfully completed the treatment modules and participated in the telephonic posttreatment assessment conducted by an independent assessor. One patient with PD who could not be assessed over the phone received all symptom rating scales via mail except for the Panic Disorder Severity Scale (PDSS).

### Inclusion Criteria

Inclusion criteria were (1) age between 18 and 65 years, (2) a primary diagnosis of PD or SAD according to the Mini-International Neuropsychiatric Interview [25], (3) moderate symptoms of PD or SAD: PDSS total score >9 or Liebowitz Social Anxiety Scale (LSAS) total score >70 [26-29], and (4) the ability to send emails and access the e-learning system.

### Exclusion Criteria

Exclusion criteria were (1) psychosis and bipolar disorder, (2) current high risk of suicide, (3) substance abuse or dependence diagnosed within the past 12 months (antisocial personality disorder), and (4) IQ<80 measured by the Japanese Adult Rating Test [30].

### Primary Outcome Measure

The primary outcome was a change in anxiety symptoms assessed using the Japanese version of the State-Trait Anxiety Inventory-Trait (STAI-T) [31]. The STAI-T is a self-reported scale comprising 40 items that quantitatively measures anxiety and can be evaluated using 2 scales [32]. The STAI-T comprises 20 items rated on a 4-point scale, and the total score ranges from 20 to 80, divided into 5 levels (I=very low, II=low, III=normal, IV=high, and V=very high). The validity and reliability of the Japanese version of the STAI-T have been demonstrated [33].

### Secondary Outcome Measures

In addition to the primary outcome, several secondary outcomes with established reliability and validity were selected based on the research objectives. The Beck Anxiety Inventory (BAI) was used to quantify comprehensive anxiety symptoms [34]. The BAI is a 21-question multiple-choice self-report inventory used to assess anxiety severity [34,35]. Other secondary outcome measures included the PDSS for participants with a primary PD diagnosis [26,27] and the LSAS for those with a primary SAD diagnosis [28,29].

The PDSS is a 7-item clinical interview rating scale that evaluates the core PD characteristics and demonstrates good psychometric properties [36]. As the PDSS is meant to be administered by professionals knowledgeable of the clinical manifestations of PD, an independent assessor (SH) was used to conduct and interpret this scale. The treatment response and remission rates were calculated using the LSAS and PDSS. For SAD, treatment response was defined as a 31% or greater reduction in the total LSAS score, and remission was defined as a final LSAS score of ≤36 [29]. For PD, treatment response was defined as a 40% or greater reduction in the total PDSS score, and remission was defined as a final PDSS score of ≤5 [37].

The trial also assessed the psychological bond between therapists and participants using the Working Alliance Inventory-Short Form (WAI-SF) [38], depressive symptoms using the Patient Health Questionnaire–9 (PHQ-9) [39,40], and generalized anxiety symptoms using the Generalized Anxiety Disorder–7 (GAD-7) [39,41]. The WAI-SF assesses the strength of the therapeutic alliance between the therapist and patient through 12 items rated on a scale of 1 (never) to 7 (always). The total score ranges from 12 to 84, reflecting the overall strength of the therapeutic bond between the therapist and patient.

Both the PHQ-9 and GAD-7 were scored on a 4-point scale (0=none, 1=a few days, 2=more than half, and 3=almost daily). The PHQ-9 scores range from 0 to 27, with the cutoff value for clinically significant depressive symptoms set at 10. Symptomatology was categorized as follows: 0-4=none, 5-9=mild, 10-14=moderate, 15-19=moderate to severe, and 20-27=severe depressive state. The Japanese version of the PHQ-9 has demonstrated adequate validity [40]. GAD-7 scores range from 0 to 21, with a cutoff value for clinically significant generalized anxiety set at 10. Symptomatology was defined as follows: 0-4=none, 5-9=mild, 10-14=moderate, and 15-21=severe general anxiety. The GAD-7 has demonstrated good reliability and validity [42].

### Intervention

We developed ICBT programs based on the Clark and Wells [43] model for SAD and Seki and Shimizu’s model for PD [44]. Each ICBT program consisted of 12 modules. Therapeutic guidance was provided by a clinical psychologist (KM) with extensive experience in face-to-face CBT for patients with PD and SAD. The therapist, a clinical psychologist with a PhD, had completed the CBT training course [45]. After each session, the therapist conducted peer supervision with the third author. The intervention involved guided ICBT comprising 12 sessions, including CBT text, comprehension confirmation tests, and explanatory videos about cognitive behavioral models, accessible through a website. The therapist guided the participants in accessing the ICBT program and answering their questions using a chat tool.

The ICBT program for PD included the following modules: guidance for ICBT program (session 0), psychological education and case conceptualization (week 1), review of safe behaviors (week 2), modification of self-image (week 3), attention shift training (week 4), behavioral experiments with internal sensory exposure (week 5), staged exposure (week 6 and 7), intervention on memory of the first panic attack (week 8), examination of others’ interpretations of panic attacks (week 9), stop dwelling on the panic attacks (week 10), reconstruction of remaining beliefs (schema work; week 11), and relapse prevention (week 12).

The ICBT program for SAD included the following modules: guidance for ICBT program (session 0), psychological education and case conceptualization (week 1), examination of safety behavior (week 2), video feedback (week 3), attention shift training (week 4), behavioral experiment 1 (week 5), behavioral experiment 2 (week 6), creation of anxiety hierarchy form and graded exposure (week 7), validating negatively rated expectations (week 8), stop dwelling on things (week 9), rewriting the meaning of memories linked to self-image (week 10), reconstruction of remaining beliefs (schema work; week 11), and relapse prevention (week 12).

The participants in this study were generally instructed not to initiate, discontinue, or modify their pharmacotherapy after the intervention. The participants were required to inform the research team if there were any changes to their medication regimen.

### Hardware and Software

The participants used personal PCs, tablet PCs, or smartphones. The software used included LearningBox (Tatsuno Information System Co) and MediLine (ShareMedical Co), a medical chat service for delivering ICBT. LearningBox is an e-learning system enabling administrators to create and manage educational materials, handle member groups, and record and assess grades. Although e-learning systems store and manage user results, they do not include personal or sensitive information.

MediLine is a medical chat service (medical social networking service) designed to replace email and phone calls. It incorporates robust encryption to prevent military-level information leaks and operates in a double-encrypted state, following the Japanese government guidelines. End-to-end encryption is performed in real time on the server during communication, and temporary memory is used in the terminal. The lectures for each CBT session were recorded as video footage, uploaded to YouTube [46,47], and shared with the participants.

### Data Setting

SH was responsible for collecting outcomes at baseline (week 1) and postintervention (week 12). The collected data were registered on a server and managed by the Data Management Office of Chiba University Hospital.

### Statistical Analysis

Statistical analyses were conducted according to the intention-to-treat principle by a team of statistical analysts (YI, YO, and YK). For screening assessments at baseline, summary statistics were generated, including proportions for categorical data and means and SDs for continuous variables. All outcomes that could be expressed as continuous variables were analyzed using paired 2-tailed *t* tests before and after the intervention. Specifically, total STAI-T, BAI, PDSS, LSAS, PHQ-9, and GAD-7 scores were analyzed. The 2-sided significance level for hypothesis testing was set at 5%, and 2-sided 95% CIs were calculated. The total WAI-SF score, which measures the strength of the therapeutic alliance, was submitted as a raw score after the completion of ICBT. One male participant with PD did not submit PDSS data after the intervention. Imputation of missing values was not performed.

## Results

### Demographic Data and Clinical Characteristics

In total, 6 participants (4 male participants and 2 female participants) with a mean age of 41 (SD 8.2; range 26-51) years were enrolled in the clinical trial. Table 1 shows the demographic data and the participants’ clinical characteristics. All participants continued their pharmacotherapy during the trial with specific medications such as paroxetine hydrochloride hydrate (n=3), venlafaxine hydrochloride (n=1), sertraline hydrochloride (n=1), and a combination of sertraline hydrochloride and ezilam (n=1). The participants’ estimated IQs tended to be higher than the mean (110, SD 4.2).

**Table 1:** Demographic characteristics.

	Participant	Participant 2	Participant 3	Participant 4	Participant 5	Participant 6
Sex	Male	Male	Female	Male	Female	Male
Age (years)	37	44	26	51	40	48
Education (years)	16	12	9	16	14	14
Employment status	Full time	Full time	Unemployed	Full time	Unemployed	Unemployed
Age at onset (years)	16	20	12	51	40	38
Primary diagnosis	SAD^a^	SAD	SAD	PD^b^	PD	PD
Comorbidity	None	None	MDD^c^	AP^d^	AP	AP
Estimated IQ	110	104	108	110	112	116

^a^SAD: social anxiety disorder.

^b^PD: panic disorder.

^c^MDD: major depressive disorder.

^d^AP: agoraphobia.

### Adverse Events

No mental or physical adverse events were reported after the intervention.

### Evaluation Outcomes

Table 2 provides a detailed overview of the outcomes. In the primary outcome, the mean total score on the STAI-T decreased by 11.0 (SD 9.6) points, although this did not reach statistical significance (95% CI –22.2 to 0.20; *P*=.05; Hedges *g*=0.95). However, several secondary outcomes demonstrated significant improvements. The mean total BAI score significantly decreased by 15.7 (SD 12.1) points (95% CI –28.4 to –3.0; *P*=.03; Hedges *g*=–1.24). The mean total PDSS score also decreased significantly, by 12.0 (SD 4.2) points (95% CI –50.1 to 26.1; *P*=.16; Hedges *g*=1.79). Furthermore, the mean total LSAS score significantly decreased by 32.0 (SD 11.1) points (95% CI –59.7 to –4.3; *P*=.04; Hedges *g*=1.38).

**Table 2:** Outcomes from pre- to postintervention (N=6).

Scale	Preintervention, mean (SD)	Postintervention, mean (SD)	*P* value
STAI-T^a^	56.8 (12.5)	45.8 (10.5)	.05
BAI^b^	34.2 (13.9)	18.5 (11.1)	.03
PDSS^c^ (n=3)	15.3 (6.03)	3.0 (4.24)	.16
LSAS^d^ (n=3)	88.3 (17.0)	56.2 (28.2)	.40
PHQ-9^e^	12.5 (5.61)	8.7 (5.61)	.16
GAD-7^f^	9.17 (6.85)	6.0 (4.5)	.27

^a^STAI-T: State-Trait Anxiety Inventory-Trait.

^b^BAI: Beck Anxiety Inventory.

^c^PDSS: Panic Disorder Severity Scale.

^d^LSAS: Liebowitz Social Anxiety Scale.

^e^PHQ-9: Patient Health Questionnaire–9.

^f^GAD-7: Generalized Anxiety Disorder–7.

Regarding depressive symptoms, the mean total PHQ-9 score decreased from 12.5 (SD 5.61) to 8.7 (SD 5.61) points, but the reduction was not statistically significant (95% CI –9.3 to 1.6; *P*=.13; Hedges *g*=0.68). Similarly, for generalized anxiety symptoms, the mean total GAD-7 score decreased from 9.17 (SD 6.85) to 6.0 (SD 4.47) points, but the reduction was not statistically significant (95% CI –9.8 to 3.5; *P*=.27; Hedges *g*=0.55).

At the postintervention assessment (week 12), the treatment response rates were 67% (n=2) for PD and 67% (n=2) for SAD. The remission rates were 67% (n=2) for PD and 33% (n=1) for SAD.

### Acceptance of Guided ICBT

Of the 4 male participants, 3 completed all treatment modules. These 3 participants completed the treatment without asking questions to the therapist and were able to understand the content and execute therapeutic tasks independently. A female participant with SAD avoided even minimal communication with therapists through chat tools due to severe social anxiety symptoms. While she accepted the self-help format of CBT, the chat with the therapist itself became burdensome rather than beneficial. A female participant with PD and fear of panic attack reported being encouraged by the therapist’s empathy and encouragement. A male participant with PD self-discontinued the treatment module and subsequently lost contact with the therapist. He frequently struggled to control his emotions, making it challenging to address his concerns effectively. For a brief case series of guided ICBT, please refer to Multimedia Appendix 1.

## Discussion

### Principal Results

This study explored the feasibility of guided ICBT in outpatients with PD and SAD in Japan. In total, 5 of the 6 participants successfully completed all modules, and no adverse events were reported throughout the study period. While anxiety symptoms decreased in most patients after the intervention, with a substantial effect size, the primary outcome (STAI-T) did not show a significant difference. However, significant improvements were observed in the secondary outcome, as measured by the BAI. Our results suggest that guided ICBT has a positive impact on anxiety symptoms, as evidenced by the notable changes in BAI scores. Most participants diligently engaged with the ICBT programs. Meanwhile, patients who considered themselves to have enough understood the materials understood the materials hardly contacted the therapists.

### Limitations

When interpreting our study, it is important to consider the following limitations. First, the small sample size prevented us from determining whether the findings were due to a type II (β) error. Second, this was a single-arm trial without a control group. More robust and statistically rigorous results could be obtained through a clinical randomized controlled trial in which the sample size is determined based on the effect sizes observed in our study. A randomized controlled trial in Japan would allow for better investigation of the effectiveness of ICBT in Japanese patients with anxiety disorders.

Additionally, we did not assess the extent to which the participants implemented ICBT program strategies in their daily lives. While the therapist encourages participants to practice what they learned through chat tools, phone calls, and in-person interactions, patients might face challenges in implementing these changes or coping with symptoms [48]. In this study, patients with PD had difficulty applying learned techniques in daily life. Future studies should evaluate the impact of practice on symptom improvement to gain a more comprehensive understanding of the therapeutic processes.

### Comparison With Prior Work

Our results showed that participants engaged in video lectures explaining the treatment modules, potentially enhancing their understanding and motivation for treatment. Despite the small sample size, guided ICBT with these lecture videos showed significant effectiveness as reflected in a treatment response rate of 67% (n=4) and a remission rate of 50% (n=3). These results are consistent with a representative meta-analysis examining treatment response predictors in 1162 patients undergoing ICBT [9]. The reduction in the total score on the primary symptom rating scales before and after the trial, along with the effect size, underscored the efficacy of guided ICBT that incorporated lecture videos. Notably, the within-group effect sizes for the primary symptoms in this study (Hedges *g*=1.38 for LSAS and 1.79 for PDSS) were as substantial as those observed in previous representative studies [49-51].

In this study, the dropout rate was 17% (1 of 6 participants), which is comparable to face-to-face CBT (26%) [52]. In contrast, another study of guided ICBT reported an average dropout rate of 32% for depression [53]. Notably, one dropout from our trial was a male patient with a significantly lower therapeutic alliance score (12 points on the WAI-SF total score). Consistent with previous studies on dropout predictors, patients with low therapeutic alliance scores tended to drop out [54]. When the therapeutic alliance is weak, therapists and patients should engage in discussions regarding treatment goals and tasks. However, rebuilding therapeutic alliances in guided ICBT contexts may be more challenging than in face-to-face contexts.

Participants with anxiety disorders might willingly participate in CBT programs but may not always practice cognitive behavioral skills [8]. For instance, one participant with PD (participant 5) experienced severe panic symptoms related to employment status. Conversely, some patients demonstrate independent therapeutic progress without extensive therapist interactions. Overall, our findings align with the existing research, indicating that severe anxiety, coexisting depression, and low socioeconomic status are associated with poor outcomes [55]. Please refer to Multimedia Appendix 1 for details regarding each participant’s treatment course.

In the case of SAD, all 3 participants successfully applied the acquired skills to their daily lives. However, none of the participants with SAD used a communication tool with their therapist. This suggests that using a chat tool may be burdensome for individuals with social anxiety, potentially reducing the accessibility of ICBT, as these patients often dislike high-intensity communication. However, the therapists’ guidance may have encouraged them to engage in self-help activities [56]. For patients with social anxiety who find it challenging to ask questions or express their thoughts, therapists could consider expressing their ability to provide answers as needed instead of engaging in routine email interactions.

It is worth noting that even patients with social anxiety who avoid chatting may still be willing to receive treatment, as indicated by a guided ICBT trial conducted in China, in which patients with SAD completed more modules of the treatment program than the nonclinical group [15]. However, if the symptoms are severe, patients may struggle to practice cognitive behavioral skills independently in their daily lives. Participants who showed symptom improvement had relatively high social functioning, continued employment, and direct community involvement (participants 1, 2, 4, and 6).

Conversely, patients with severe disorders (participant 3) and those resistant to exposure-mediated interventions (participant 5) may require a significant amount of therapeutic attention. For instance, individual face-to-face CBT might be a more suitable option for someone like participant 3, who has SAD. Similarly, for participant 5, a personalized face-to-face CBT approach in which the therapist helps analyze the gains and losses of exposure and safety behaviors and demonstrates internal sensory exposure techniques could be beneficial. Clinicians and therapists who are going to conduct CBT should consider disclosing treatment modules to patients and assess the level of support required before providing ICBT.

### Conclusions

Guided ICBT may be a feasibility treatment approach for patients with PD and SAD in Japan. If the patients have high motivation and the ability to understand and practice treatment modules, supports might not be strictly necessary during the treatment from the CBT therapist. The empathetic words from the therapist and encouragement for program implementation may assist some patients in overcoming fear and engaging in the assigned tasks.

## Appendix

Multimedia Appendix 1 Case series of the 6 participants.

## Abbreviations

BAI: Beck Anxiety Inventory
CBT: cognitive behavioral therapy
GAD-7: Generalized Anxiety Disorder–7
ICBT: internet-based cognitive behavioral therapy
LSAS: Liebowitz Social Anxiety Scale
PD: panic disorder
PDSS: Panic Disorder Severity Scale
PHQ-9: Patient Health Questionnaire–9
SAD: social anxiety disorder
STAI-T: State-Trait Anxiety Inventory-Trait
WAI-SF: Working Alliance Inventory-Short Form

## Data Availability

The data sets generated and analyzed during this study are available from the corresponding author on reasonable request.
