# Supplementary material for: Feasibility of Guided Internet-Based Cognitive Behavioral Therapy for Panic Disorder and Social Anxiety Disorder in Japan: Pilot Single-Arm Trial
Source: JMIR Form Res. 2024 Feb 29;8:e53659. doi: 10.2196/53659 (PMC10940979; doi:10.2196/53659)
Supplement: Multimedia Appendix 1 [file formative_v8i1e53659_app1.docx]

## **Case Participant 1: Male with Social Anxiety Disorder**

This case involved a 37-year-old man employed in the delivery industry. Initially, participant 1 expressed fear about reporting to his boss and believed that his body odor was excessively offensive to those around him. He actively participated in the program right from the start and successfully completed all 12 modules. During the course, he identified his safety behaviors, such as wearing sunglasses and avoiding eye contact, and gradually exposed himself to various social situations. He gained knowledge and skills rooted in cognitive behavioral science and applied them in his daily life without requiring direct interaction with the therapist. Upon finishing all the modules, he reported a significant reduction in his social anxiety symptoms. He provided feedback stating that the online treatment experience was easy to comprehend and aided him in developing new cognitive and behavioral techniques.

## **Case Participant 2: Male with Social Anxiety Disorder**

This case involved a 44-year-old man employed as an engineer in a factory. Since starting this job, he experienced excessive nervousness during meetings, especially when asked for his opinion. Upon receiving a promotion shortly before joining the trial, he found himself interacting more frequently with colleagues and subordinates. Driven by a strong motivation to overcome his social anxiety at work, he actively engaged in all treatment modules and diligently practiced cognitive behavioral skills.

A particularly effective strategy for him was to abstain from safety actions, such as avoiding eye contact with coworkers and refraining from communication. He approached speaking at meetings as a behavioral experiment, successfully challenging his negative beliefs about social anxiety through unexpectedly positive experiences. His social anxiety symptoms improved as he gradually exposed himself to interpersonal situations at the workplace that he had previously avoided. Remarkably, this improvement was primarily self-driven. Right from the start, participant 2 chose not to use chat tools to communicate with the therapist. The therapist conducted interviews with him at an outpatient clinic, inquiring about his progress.

Participant 2 provided positive feedback on the program content, stating, "I had no problems implementing this program on my own." Despite the option of transitioning to face-to-face sessions after completing the ICBT program, he declined due to time constraints at work.

## **Case Participant 3: Female with Social Anxiety Disorder**

This case involved a 26-year-old woman who struggled with social withdrawal and experienced both depression and social anxiety at the time of screening. She rarely left her home due to extreme nervousness in interpersonal situations, even wearing a mask and hat when she had to visit the hospital. With an understanding of the cognitive behavioral model of social anxiety, she gradually reduced her safety behaviors and practiced attention shifts.

Communicating with the therapist posed a significant challenge for her, and she never utilized medical chat tools during the trial. Despite this, she managed to complete the ICBT program independently. During a meeting with one of the researchers at a hospital post-intervention, she mentioned that although she couldn't interact with the therapist through chat or telephone, she successfully completed the program on her own.

When the therapist suggested the possibility of face-to-face sessions to further improve her symptoms, she expressed her inability to tolerate such interactions, stating, “It’s been a little easier in social situations, but it is intolerable for me to conduct face-to-face sessions." In response, her therapist assured her that the ICBT program would remain available for her use. And face-to-face CBT could be arranged whenever she felt ready. She thanked the therapist and expressed that the self-help ICBT format was just right for her.

## **Case Participant 4: Male with Panic Disorder**

This case involved a 51-year-old building company employee who participated in the clinical trial due to panic attacks triggered by a traffic accident. During the screening, he exhibited mild panic symptoms and agoraphobia but did not have posttraumatic stress disorder. His safety behaviors included focusing on his breath and pulse in the car, stopping when he felt anxious, using specific routes like highways, and being aware of his body sensations.

He actively engaged in learning skills rooted in cognitive behavioral science to overcome his panic symptoms. The confirmation test held significant importance for him, as he mentioned, “If I thought I understood [the skills] well, I [took the test and] got at least 50% of the answers on the first confirmation test correct.” He diligently reviewed the program multiple times, provided feedback, and continued with confirmation tests until he achieved a 100% correct answer rate. He applied what he learned in his daily life, particularly finding it helpful to shift his attention to external stimuli when he noticed himself focusing on internal sensations while driving.

Interestingly, he did not require chat tools to communicate with the therapist because his confirmation test demonstrated a strong grasp of CBT, enabling him to effectively manage his panic disorder. Instead, the therapist communicated with participant 4 over the telephone. During these conversations, he consistently reported, “It’s going well. It’s easier to go out than ever before. I’m sorry I did not contact you.” Following the completion of the treatment, he informed the therapist, “I didn't feel it was necessary to contact you because I wasn’t experiencing any problems.”

## **Case Participant 5: Female with Panic Disorder**

This case involved a 40-year-old full-time housewife raising an infant who was struggling with panic attacks and agoraphobia during the screening. After initiating the program, she successfully grasped her cognitive and behavioral patterns and identified her safety behaviors. Session 5 introduced internal sensory exposure, which was recommended to be practiced daily for eight weeks after the session. However, she was hesitant to engage in behavioral experiments, especially internal sensory exposure, conducting them only a few times in the latter half of the ICBT program due to her intense fear of palpitations.

Gradual exposure to agoraphobia had a positive impact on her, likely due to the necessity of going out with her child. Despite persisting with safety behaviors such as measuring her pulse thrice a day and some rumination habits, she acknowledged an improvement in her panic and agoraphobia symptoms after the intervention. The empathy she received from the therapist through the chat tool provided her with a sense of calm and encouragement, giving her the courage to tackle exposure tasks. She reported feeling more motivated to continue treatment, recognizing that she was on the right path to alleviate her conditions.

## **Case Participant 6: Male with Panic Disorder**

This case involved a 48-year-old man who had retired due to panic disorder and was experiencing panic symptoms and agoraphobia during the screening. His fear of suffocation led him to avoid exercise, cooking, and going out alone. He had a comprehensive understanding of his safety behaviors and panic symptoms. The therapist suggested interoceptive exposure methods such as breathing through a straw or climbing stairs, but he did not engage in any behavioral experiments involving exposure. Starting from session 5, which focused on behavioral experiments, he did not participate in any programs. He frequently struggled to control his emotions, making it challenging to address his concerns effectively. The therapist attempted to understand his feelings and engage in constructive dialogue. However, due to irregular contact, they couldn't establish consistent communication. Consequently, the patient did not feel adequately supported and eventually discontinued his treatment.
